# Decreasing the number of false positives in sequence classification

**DOI:** 10.1186/1471-2164-11-S5-S10

**Published:** 2010-12-22

**Authors:** Ariane Machado-Lima, André Yoshiaki Kashiwabara, Alan Mitchell Durham

**Affiliations:** 1 Escola de Artes, Ciências e Humanidades, Universidade de São Paulo, Rua Arlindo Béttio, 1000, 03828-000, São Paulo, SP, Brazil; 2 Instituto de Psiquiatria, Universidade de São Paulo, R. Dr. OvÍdio Pires de Campos, 785, 01060-970, São Paulo, SP, Brazil; 3 Instituto de Matemática e EstatÍstica, Universidade de São Paulo, Rua do Matão, 1010, 05508-090, São Paulo, SP, Brazil

## Abstract

**Background:**

A large number of probabilistic models used in sequence analysis assign non-zero probability values to most input sequences. To decide when a given probability is sufficient the most common way is bayesian binary classification, where the probability of the model characterizing the sequence family of interest is compared to that of an alternative probability model. We can use as alternative model a *null model*. This is the scoring technique used by sequence analysis tools such as HMMER, SAM and INFERNAL. The most prevalent null models are position-independent residue distributions that include: the uniform distribution, genomic distribution, family-specific distribution and the target sequence distribution. This paper presents a study to evaluate the impact of the choice of a null model in the final result of classifications. In particular, we are interested in minimizing the number of false predictions in a classification. This is a crucial issue to reduce costs of biological validation.

**Results:**

For all the tests, the target null model presented the lowest number of false positives, when using random sequences as a test. The study was performed in DNA sequences using GC content as the measure of content bias, but the results should be valid also for protein sequences. To broaden the application of the results, the study was performed using randomly generated sequences. Previous studies were performed on aminoacid sequences, using only one probabilistic model (HMM) and on a specific benchmark, and lack more general conclusions about the performance of null models. Finally, a benchmark test with *P. falciparum* confirmed these results.

**Conclusions:**

Of the evaluated models the best suited for classification are the *uniform model* and the *target model*. However, the use of the *uniform model* presents a GC bias that can cause more false positives for candidate sequences with extreme compositional bias, a characteristic not described in previous studies. In these cases the target model is more dependable for biological validation due to its higher specificity.

## Background

Probabilistic models are widely used in biological sequence analysis. They are essential mechanisms to pre-process the plethora of data available, creating hypothesis for biological validation. Examples are Hidden Markov Models (HMM) [1-3], Weight Array Matrices (WAMs) [4] and Covariance Models (CMs) [5]. In this context, probabilistic models can be used to represent known families of sequences and to create programs to predict if specific sequences belong to the family of interest. However, these models assign non-zero probability values to most input sequences. Therefore, we need a criteria to decide when a given probability value is sufficient. One of the most commonly used technique is bayesian classification using two probabilistic models: *F*, that represents a family of sequences, and *A*, an *alternative model*. The likelihoods of each of the two models is measured and the sequence is classified as belonging to *F* if the likelihood of *F* is greater than the likelihood of *A*.

The choice of the alternative model is essential to reduce the number of false predictions and depends on the problem. An alternative model can be either a *negative model* representing the complementary set of the sequences of interest, or a *null model*, representing random sequences. Negative models are used when there is a deeper biological understanding of the particular problem and it is possible, with a high degree of certainty, to characterize the sequences that are not part of the family. Therefore, the choice of the probabilistic model to be used as the negative model depends on a strong biological hypothesis about the complementary set. Null models are used when we do not have sufficient information to characterize the complementary set of the sequences we want to classify. This situation is generally the rule for annotation software, where we want to characterize a sequence family (e.g. tRNAs, exons, miRNAs, transmembrane domains,...) against *all* other sequences. This is the scoring technique used by sequence analysis tools such as HMMER [2], SAM [6] and INFERNAL [7]. Null models is the chosen strategy for alternative model considered in this work.

More technically, we want to compute, given a nucleotide sequence *x,* which model better represents the sequence: the family model (representing the family of sequences we are interested on) or the null model (representing other sequences). The sequence *x* is classified as belonging to the family represented by the model *F* if *P*(*F|x*) *> P*(*N|x*) or, alternatively, if . Considering *P*(*F*) *= P*(*N*)*,* the classification of *x* simplifies to the comparison of the likelihoods:  . To cope with the very small probability values when sequences are long, log values are used. So, we use the *log-odds* score *S:*(1)

We want null models that help classifiers reject sequences that do not belong to family *F* (which we will call *negative sequences*)*.* Therefore, such a null model *N* should score higher than the family model *F* for any negative sequence. In other words, with a good null model, log-odds score for negative sequences will have value zero or less.

Null models, due to their very generic nature, should not present any structure. Therefore a convenient model to describe random sequences in a null model *N* is a position-independent probability distribution, which imposes no structure on the sequences. For nucleic acids sequences, the null model assigns a fixed probability value *P_N_*(*i*) to each nucleotide (*i = A, C, G, T*)*.* Therefore, the probability value of a sequence *x* of length *L* is given by the formula:

*P*(*x*|*N, L*) = [*P_N_*(*A*)]^c_A_^*[*P_N_*(*C*)]^c_C_^*[*P_N_*(*G*)]^c_G_^*[*P_N_*(*T*)]*^c_T_^*(2)

where *c_i_* is the count of the nucleotide *i* in the sequence *x*, .

There are many possible strategies to set up a null model discussed in literature [8-10], all of which seem to make sense biologically. Some of them are: i. using a uniform distribution, ii. using the genomic background distribution, iii. using the training set distribution, iv. using the target sequence distribution. Each strategy uses a different reasoning to minimize false positives. The reasoning behind each strategy is based on how we will characterize "random sequences". With the uniform distribution, we define randomness by the absence of information, even about the nucleotide composition. With the genomic background distribution, we define randomness by what should be the standard nucleotide distribution of a sequence in a specific genome. With the training set distribution, we assume the family model will favor a certain specific nucleotide distribution (that of the known sequences of that family used to infer the model, the training set); so if we use the nucleotide distribution of the training set as a null model, this will help the classifier reject sequences with a high score only due to their base composition. Finally, with the target sequence distribution, random sequences are those with the same base distribution of the target sequence (in other words, a genomic background strategy reduced to the sequence locality). Independently of the rationale chosen, the null model will fall in one of three classes: a uniform distribution, a fixed non-uniform distribution or a target-dependent distribution.

The goal of this study is to evaluate the impact of each of these three classes of null models in the false positive rate of classifiers. We found only two studies in literature that analyzed the performance of null models [8,9]. Each study evaluates one specific benchmarks of aminoacid sequences and only one probabilistic model (HMM). This approach limits the generality of their conclusions. First, they do not address the problem for a wider amplitude of classification models. Second, and more important, they only analyze the final accuracy results for their specific benchmarks, without any consideration on why these can be generalized to other sequence families.

To make this study more general than previous works, we use random sequences and two different probabilistic models. Using random sequences guarantees there is no bias in the study towards any particular benchmark, so we expect the results to be of broad application. Also, the simulations used random sequences across the whole GC spectrum, in an effort to make the results applicable to any real-life situation. The two probabilistic models chosen are very different, aimed at covering a wide range of models: one with very simple architecture and one able to represent more structured sequences. The studies were performed using Weight Array Matrices (WAMs) [4] and Covariance Models (CMs) [5].

WAMs record only fixed-distance content dependencies, useful to represent sequence motifs. CMs are able to characterize indels and register dependencies in non-adjacent bases at arbitrary distances, which can be used to characterize secondary structure. We evaluated WAMs in the context of splice site prediction and CMs in the context of predicting RNA or other genomic elements with secondary structure. Splice sites were used for three reasons: first, splice site prediction is at the heart of gene prediction, an biologically important problem in bioinformatics, second, the abundance of data in public databases, third, because many successful predictors use position-dependent models, which is the base of our probabilistic model range. The spectrum of GC content in the dataset enabled using a single sequence family (splice sites) for all experiments with WAMs. In this context, the same was not possible for CMs, where training sets are generally small and concentrated on a small spectrum of GC content. In this case we had to use three different sequence families (see methods for details).

We will see below that the training set and the genomic background are not good choices for a null model. In fact, no fixed, non-uniform distribution is, as a quick mathematical analysis can demonstrate. As we will see below, two probabilistic i.i.d. models are best suited for classification: the *uniform model* and the *target model*. However, we also show that the uniform distribution can also have a deleterious effect in sequences with biased GC distribution. This is particularly relevant, since it has not been described before and since uniform models are widely used in the context of nucleotide sequences. The final conclusion is that the target model is more dependable when choosing candidates for biological validation due to its higher specificity. This is reinforced by the real data experiment using *Plasmodium falciparum*, a highly AT-rich genome. The study was performed in DNA sequences using GC content as the measure of content bias, but the results should be valid also for protein sequences.

## Results

Since we are interested in minimizing the number of false positive predictions, we used randomly generated sequences for evaluation. Random sequences should receive negative log-odds scores in probabilistic classifiers for any specific sequence family. In other words, a better performance in terms of specificity means fewer random sequences with positive scores. We evaluated six null models: 5%GC, 25%GC, uniform, 75%GC, 95%GC and the model obtained from the base frequencies in the target sequence (the target model)^1^.

^1^We used GC content as a simplified measure of nucleotide composition, which allows the visualization of 2D plots.

Initially, for illustrative purposes, we computed the log of the probability values of the test sequences given the null models alone (no log-odds score). This illustrates the values produced by these models for sequences at different GC compositions. We called these “raw scores”. Next, we used each of these models as null models in log-odds scoring classification for two different types of family models, WAMs and CMs. Since we are using only random sequences, the log-odds scores should be negative. Positive scores indicate false positives^2^.

^2^We assume that the chance of one of the random sequences being an actual family sequence is negligible.

### Raw score behavior on random sequences

**Figure 1:**
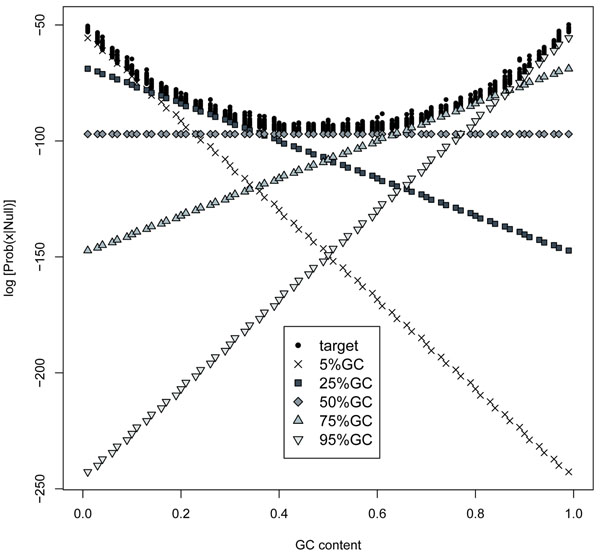
**Raw scores of random sequences** Raw scores (log of the probability given the null model) of random sequences as a function of their GC content. The raw scores were calculated using the six models: • target model (with the nucleotide distribution of the analyzed sequence) and 5 fixed GC models (x: 5%, □ 25%,◊: 50%, Δ: 75% and ∇ 95%).

We have plotted the raw scores (log of the probability value) of random sequences using the fixed distribution and target models alone. The results are shown in Figure 1.

As it was expected, the uniform model produces no bias along the GC content (x axis), producing a constant score, consistent with the fact that all analyzed sequences have the same size. The raw scores using the biased fixed distribution models (5%GC, 25%GC, 75%GC, 95%GC) show a linear dependence on the GC content of the analyzed sequences; the GC content of the model only determines the inclination of the linear plot. The target model presents a less intuitive result, a curve with the lowest scores at 50%GC and higher scores towards more extreme GC distributions.

### Effect of different null models in log-odds scoring

Probabilistic models such as WAMs and CMs also capture the base composition of the sequences of the training set. Therefore, when we use log-odds scoring, the GC bias recorded by the family models should also influence the final score and we have to analyze the combined influence of the family and null models. We embedded the null models used in the previous section in classifiers using two different probabilistic techniques, Weight Array Matrices (WAMs) [4] and Covariance Models (CMs) [5]. For each technique we created classifiers using training sets with different GC average compositions. For clarity, we only show the results for 4 null models: target, 5%GC, 50%GC and 95%GC. Data for the null models corresponding to the 25%GC and 75%GC is consistent with the presented results (data not shown).

#### Weight array matrices

**Figure 2:**
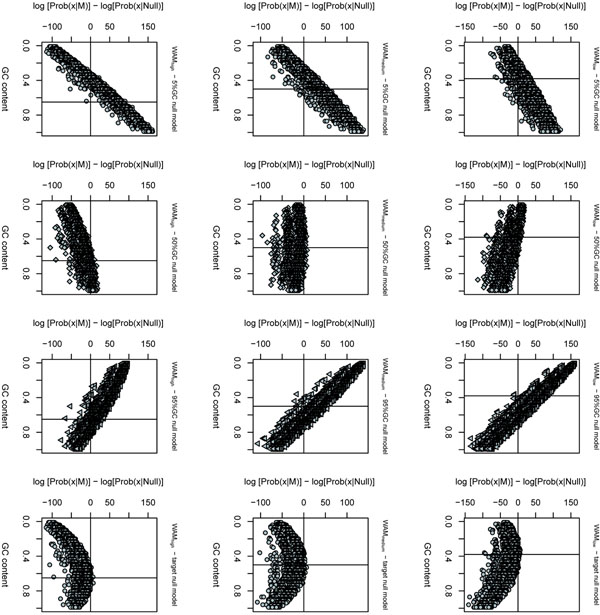
**Log-odds scores of weight array matrices** Null model influence on log-odds scores based on weight array matrices (WAMs). The plots show the log-odds scores of random sequences as a function of their GC content. In each plot, a horizontal line indicates the 0 log-odds score (used as classification threshold) and a vertical line indicates the average GC content of the training sequences. WAMs inferred by low, medium and high GC training sequences were used in the plots at lines 1, 2 and 3, respectively. 5%GC, 50%GC, 95%GC and target null models were used in the plots at columns 1, 2, 3 and 4, respectively. Note: the three bands visible in the plots are generated by the WAM models, and not by the use of any particular null model.

We used sequences of acceptor splice sites to create three distinct training sets with different average GC content: 38%GC, 50%GC and 65%GC (see Materials and Methods for a justification on GC percentages). For each training set, a weight array matrix was trained and used to score random sequences using the six null models: 5 fixed GC models and the target model. The results are shown in Figure 2.

As we can see, log-odds scores of random sequences using fixed GC null models, including the uniform model, present a quasi-linear dependence on their GC content. This means that, no matter what is the composition of the sequences used to characterize the family (the training set), any random sequence at the ends of the GC spectrum will score consistently higher (or lower) than any other sequence. This effect is so relevant that random sequences in one of the GC content extremes have positive scores when any of the fixed GC models is used, which indicates a strong tendency to generate false positives in the classification of sequences with extreme GC compositions. On the other hand, the target null model presents higher scores for sequences with GC content similar to the average GC content of the training set and lower scores for sequences with extreme GC content. The target null model presents the lowest number of positively scored sequences. The consequence in real-life classifications would be a lower number of false positives.

#### Covariance Models

Covariance Models (CMs) are usually used to characterize families of RNAs or other genomic elements with secondary structure. Training sets for CMs tend to be much smaller. Therefore, instead of dividing the training set of a single family in different training sets separated by GC content (as performed in the analysis using WAMs), we used three different CMs obtained from the RFAM database [11], each one targeting a family with a distinct compositional bias: i. the *CM_low_* family with 5.6% GC (rbcL 5’ UTR RNA stabilizing element, rfam number RF00197), ii. the *CM_medium_* family, with 49.2% GC (small nucleolar RNA SNORA67, rfam number RF00272), iii. the *CM_high_* family, with 71.4% GC (bag-1 internal ribosome entry site - IRES, rfam number RF00222).

**Figure 3:**
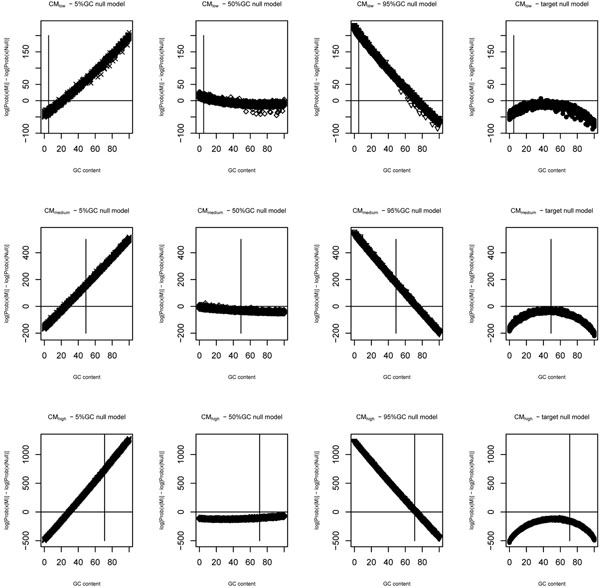
**Log-odds scores of covariance models** Null model influence on log-odds scores based on Covariance Models (CMs). The plots show the log-odds scores of random sequences as a function of their GC content. In each plot, a horizontal line indicates the 0 log-odds score (used as classification threshold) and a vertical line indicates the average GC content of the training sequences. CMs inferred by low, medium and high GC training sequences were used in the plots at lines 1, 2 and 3, respectively. 5%GC, 50%GC, 95%GC and target null models were used in the plots at columns 1, 2, 3 and 4, respectively.

We can observe in Figure 3 that log-odds scores using the fixed GC distribution null models show, again, a quasi-linear dependence on the GC content, resulting in a large number of false positives in one side of the GC spectrum. Similar to what happened with WAMs, the uniform model also shows a reduced, but still present quasi-monotonic bias (i.e. only increasing or only decreasing). The target model, on the other hand, shows a non-linear dependence on the GC content, with a peak towards the GC content of the training set.

### Specificity of the different null models

Table 1 clearly shows the superior specificity performance of the target null model over all other options. We can see that the target null model shows a significantly lower number of positive scores against the other models in almost all of the six families. The only exception was the RNA GC-rich family (*CM_high_*), where the uniform null model tied with zero false positives.

**Table 1:** Specificity of the different null models

null model	WAM_*low*_	WAM_*med*_	WAM_*high*_	CM_*low*_	CM_*med*_	CM_*high*_
5%GC	2431 (70%)	2377 (68%)	2173 (63%)	2439 (77%)	3775 (75%)	3567 (71%)
25%GC	1118 (32%)	1517 (44%)	1500 (43%)	1681 (53%)	2509 (50%)	2243 (44%)
50%GC	863 (25%)	79 ( 2%)	700 (20%)	674 (21%)	58 ( 1%)	0 ( 0%)
75%GC	1443 (42%)	1534 (44%)	738 (21%)	1644 (52%)	2521 (50%)	2197 (44%)
95%GC	2114 (61%)	2332 (68%)	2529 (73%)	2297 (73%)	3642 (72%)	3625 (72%)
target	45 ( 1%)	18 ( 0%)	25 ( 0%)	3 ( 0%)	0 ( 0%)	0 ( 0%)

Number (and percentage) of positively scored sequences for each null model. WAM_*low*_, WAM_*med*_ and WAM_*high*_ designate the WAM models generated by the training set with low (36%), medium (48%) and high (65%) GC content, respectively. CM_*low*_, CM_*med*_ and CM_*high*_ designate the CM models generated by the training set with low (5.6%), medium (49.2%) and high (71.4%) GC content, respectively.

### Testing in *Plasmodium falciparum* real data

**Figure 4:**
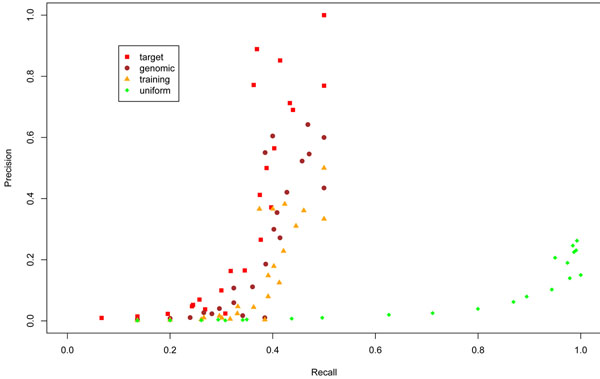
**Precision-Recall Graph for *P. falciparum* data** This picture shows the precision-recall graph for the acceptor splice site prediction in *P. falciparum* comparing four different null models on respect of different GC contents of sequences. The tested null models were the target, the uniform, the training set distribution and the genomic background null models.

As we have seen above, the target null model presented much better performance against the other models when testing against random sequences. To validate these results in a realistic environment, we have tested the performance of 4 null models in the context of acceptor site prediction for *Plasmodium falciparum*. This organism was chosen due its well known GC bias (19% GC). We tested four null models used in prediction: target null model, uniform null model, genomic background null model (19% GC) and training set null model (17.9%GC). The precision-recall curves are depicted in Figure 4. As expected, splice site prediction with the target null model shows a significantly better performance, with a higher precision (percentage of true prediction among the positive results) in most marks. Considering all the tested sequences (all the GC marks) the target null model presented the best precision (22.81%), the best specificity (99.12%), and the best balance between precision and sensitivity (F-score = 28.16%) (Table 2). A ROC curve has also been generated and reinforced the best performance of the target null model (Additional file S1).

**Table 2:** Performance of the null models on the *P. falciparum* data

	Precision	Specificity	Sensitivity	F-score
target	**22.81%**	**99.12%**	36.79%	**28.16%**
uniform	3.51%	84.39%	**81.28%**	6.74%
genomic	13.07%	98.18%	39.01%	19.58%
training	5.39%	95.24%	38.69%	9.46%

This table shows the precision, specificity, sensitivity, and F-score for the entire testing set of the *P. falciparum* using the target, uniform, genomic background and the training null models. The best performance for each measure is in bold face. The F-score, is the harmonic mean between precision and sensitivity values.

## Discussion

The results of raw scores presented above show that all but the uniform null model produce a score biased by the GC content of the analyzed sequence. The problematic aspect is not the GC dependence *per se*, but when this dependence produces a raw score curve linear in the whole GC spectrum. So, for instance, if the null model is 75%GC, the greater the GC content of the sequence, the higher the final raw score, including the sequences with GC content higher than 75%. On the other hand, the dependence introduced by the target model is a curve with higher values at the extreme GC contents and with the lowest value at 50%GC. So high scores will always be attained by sequences with any kind of bias (high or low GC). Therefore, raw score curves seem to indicate that the only adequate null model is the uniform model, since it is the only one that does not introduce a dependence on the GC content.

Indeed, when the models were used in log-odds scoring, the uniform model showed the lowest dependence on the GC content (Figures 2 and3), but still showed a quasi-linear dependence (albeit smaller than the other models). This indicates that the GC content registered by the family models also have a small deleterious effect when working with sequences with high GC bias, since positive scores were ascribed to some random sequences. The curve associated with the target model, on the other hand, presented a peak near the average GC content of the training sequences, “canceling” the monotonic GC-dependence introduced by the family models.

This is an interesting feature, since the GC content can be a meaningful characteristic of a sequence family. In fact, this is a more appropriate classifier behavior than the effect associated to other null models, such as a training set null model, that assign high scores to sequences with a GC bias opposite to that presented by the sequences of the targeted family. If a family of sequences has low compositional variation, GC content can be considered relevant information during the classification process. What we want in these cases is a dependence that will “center” at the characteristics of our training sets, that is, that rewards GC contents similar to those of the known sequences of the targeted family and that “punishes” GC contents that are not. That is exactly what the target null model does, without producing too many false positives.

The location of the “peaks” (near the training set GC content) is not a coincidence. In particular, if the family model *F* is also a position-independent fixed-distribution model, then the log-odds peak is exactly at the nucleotide distribution represented by the family model. This happens because, by definition, the target model is a distribution model trained using the target sequence with the maximum likelihood method, i.e, the probability of the sequence given the target model is the maximum value possible in the family of distribution models. So the peak observed in the log-odds curve occurs when both models have the same nucleotide distribution and it occurs when the target sequence has the same nucleotide composition as represented by the family model. In other words, when only GC composition is concerned, log-odds scoring with the target model peaks at the family’s GC content.

Also important is that the peak scores presented in the target null model do not necessarily correspond to positive scores. In fact, the target null model presented the best specificity results (lowest number of positive scores for random sequences) in all tests. Moreover, this effect is still in place even for models that register secondary structure such as the CMs. In this case, although the log-odds score peak is moved towards the average GC content of the family, they do not coincide exactly (which occurred in the WAM-based classifiers). The explanation is probably related to the structural component of the CM score, which is not so directly dependent on the sequence GC content.

If on one hand the target null model presents the best specificity, on the other hand it may impair sensitivity in detecting true sequences that have the base composition very different from the average composition of the training set. When a high GC variation is expected within the family of interest, it is possible that the target model will generate a higher number of false negatives, in which case the uniform model should also be considered. This phenomenon was observed in covariance model tests performed with a benchmark of transfer RNA sequences (tRNAs) (data not shown). For a test sample of 100 tRNA sequences^3^ with GC content evenly distributed over the GC range of the tRNA family (from 8.8% to 74.3%GC), the specificity values achieved using the uniform and target null models were, respectively, 96.7% and 100% and the sensitivity values were, respectively, 100% and 93%, corroborating the fact that the target model tends to have higher specificity and lower sensitivity than the uniform model. The same behavior was observed in the *Plasmodium falciparum* data experiment presented in this work. The target null model presented the best precision (22.81%), but its sensitivity was 36.8% (Table 2). The most sensitive model was the uniform null model (81.3%) but at the cost of a very low precision (3.5%). So in this specific context the use of the uniform null model is not recommended. We do not evaluate false negative rates, since this evaluation cannot be performed using random sequences and is, therefore, highly dependent on the benchmark used.

^3^Sequences downloaded from Rfam database release 8.1 [11] under the Rfam accession number RF00005.

The GC percentages of the fixed distribution null models shown in this article do not correspond to the specific GC contents that would constitute a “training set” null model on each experiment using simulated data. But, in fact, “training set” null models are fixed-distribution models, where the distribution is determined by the training set. Therefore, a training null model is not suitable because of its fixed distribution. The homogeneous behavior of the performance of fixed-probability null models and the inferior performance of the training null model in the real data experiment support our conclusions. Also, for the covariance models, the training set percentages (5.6%, 49.2%, 72.4%) were very close to the percentages used in the tests (5%, 50%, 75%). The same is not true for the WAM tests, in which case we did run tests for null models with the training set percentages, and the results were consistent (data not shown).

Our study was performed in the context of nucleotide sequences, however we expect similar results for aminoacid compositions. This is supported by the fact that the analytical reasoning we performed are also valid for aminoacid sequences. In other words, when using any fixed distribution model against the target null model in log-odds scoring, the highest scores are obtained for sequences with the same aminoacid composition as that described in the fixed model. Due to the number of possible aminoacids, a similar study would be harder to perform and interpret it as 2D plots would not be helpful. As a matter of fact, two HMM-based tools used for protein domain identification, SAM [6] and HMMER [2] also make use of target sequence data in some way to compose their null model. SAM scores the reversed target sequence with the same HMM. HMMER combines the database background frequencies with a second null model derived from the analysis of the target sequence in an *ad hoc* way [2]. Their success seems to reinforce our belief.

## Conclusions

In this paper we evaluated the performance of 3 different types of null models in profile-based probabilistic models: *uniform* null model, *fixednon-uniform* GC null model (5%, 25%, 75% and 95%), and target null model on the analysis of random nucleic acids sequences of various GC contents. We presented both the independent behavior of each model in the form of raw scores and their behavior when used in log-odds scoring in conjunction with 2 different probabilistic techniques: Weight Array Matrices (WAMs) [4] and Covariance Models (CMs) [5].

All our results indicate that, when the sequence family presents low variation on the GC content, the target model is a more dependable model to generate hypothesis for biological verification due to its high specificity when compared to any fixed-distribution model, in particular for organisms that present genomic sequences with high GC bias. Detecting acceptor splice sites in the GC-poor *Plasmodium falciparum* genome (19% GC), the target null model presented the best precision (22.81%), the best specificity (99.12%) and the best balance between precision and sensitivity (F-score = 28.16%). The use of the target residue composition in the null model construction was also shown beneficial in substitution score matrices. Yu, Altschul and colleagues proposed matrix modifications taking into account the amino acid frequency of both query and target sequence [13,14]. Accuracy of the PSI-BLAST program [15] is also improved by re-evaluating promising alignments using statistics based on the composition of target sequences [16].

This study was performed using 2 probabilistic techniques, WAMs and CMs. However, we expect the results to hold for other techniques such as Weight Matrix Models [17] and Hidden Markov Models [1], which are particular cases of WAMs and CMs, respectively.

## Methods

### Generation of the random sequences

The random sequences were generated by a Perl script written for this study. We wanted to visualize howthe models behave when analyzing heterogeneous sequences, i.e, sequences with different GC contents. Thus we needed a homogeneous number of sequences in each GC content mark. The script is parameterized by two values, *N* the number of sequences for each percentage mark, and *L* the length of the sequences. It generates *N* sequences for each GC percentage *W,* varying from 0.00 to 1.00 in intervals of 0.01 (101 GC marks). For each *W,* a sequence of length *L* is created by generating two subsequences: (1) one subsequence of length *K*_1_*=* ⌊L * W⌋ containing only the symbols G and C chosen from a uniform distribution; (2) another subsequence of length *K_2_ = L — K*_1_ containing only the symbols A and T chosen from a uniform distribution. The final *N* sequences are random shufflings of the sequence generated by concatenating these two subsequences. Step (1) induces a process that may generate datasets with a different number of sequences associated to each value *o*f *W.* So the datasets were pruned to ensure 0 or *N* sequences for each value of *W.* However, this process generated datasets with different total sizes (less than or equal to *N* * 101).

Five sets of random sequences were generated using the approach described above: one for the raw score computations (raw score set, 5050 sequences), one for the WAM evaluations (WAM set, 3450 sequences) and one for each of the three CMs (*CM_low_* with 3150 sequences, *CM_medium_* and *CM_high_* with 5050 sequences). Different sets were generated to accommodate the particularities of each analysis. The raw score set consists of sequences of length 100, the WAM score consists of sequences of size 70 (the size of the acceptor splice sites used to train the family model) with the AG consensus at the splice site position. Each of the three covariance sets consists of sequences with a specific length, each length corresponding to the average length of the sequences used to train each of the CMs (62nt, 164nt and 402nt for *CM_low_*, *CM_medium_* and *CM_high_* respectively).

### Obtaining raw scores for the fixed distribution and target models

We calculated the probability of the random sequences using six different probabilistic models: i. five fixed GC null models (5%GC, 25%GC, 50%GC, 75%GC, 95%GC), with G and C having the same individual probability, as well as A and T; ii. the target null model. We plotted the raw scores (logarithm of the probability value) versus the sequence GC content to illustrate the independent behavior of each null model. We show in this paper the plot using sequences with length 100. Plots for the other lengths are similar: the same curve inclinations with different score limits. To calculate the probability value of the sequence *x* given a null model *N*, we use the formula:

*P*(*x*|*N*, *L* = 100) = [*P_N_*(*A*)]^c_A_^ * [*P_N_*(*C*)]^c_C_^* [*P_N_*(*G*)]^c_G_^* [*P_N_*(*T*)]*^c_T_^*(3)

where c*_i_* is the count of the nucleotide *i* in sequence *x* and .

### Obtaining log-odds scores for WAMs

Acceptor splice sites from the HS3D database release 1.2 [18] were used to build three training sets with different average GC content. We trimmed each sequence so that the resulting sequences had length 70 with the canonical AG sequence at position 49 (equation 1). We built three distinct training sets to characterize distinct GC biases, by partitioning splice site sequences by their GC content. The three training sets were: i. **low GCcontent** containing 1013 sequences with GC content less or equal to 50% (average GC content of 38%); ii. **medium GC content** training set containing 1381 sequences with GC content between 50% and 60% (average GC content of 50% ); iii. **high GC content** training set containing 1006 sequences with GC content greater than 60% (average GC content of 65%).

For each training set, we estimated Weight Array Matrices (WAMs) [4] for acceptor splice sites using MYOP, a framework for generating probabilistic classifiers [19]. Using the estimated WAMs, we calculated the log-odds scores for the random sequences with length 70 and with a consensus AG subsequence at position 49. Two different types of null model were applied: i. five fixed GC models (5%GC, 25%GC, 50%GC, 75%GC and 95%GC); ii. target null model. We plotted the log-odds scores versus the GC content of each random sequence to visualize the influence of null models on the calculated scores.

### Obtaining log-odds scores for CMs

Three RNA families with different GC content averages were chosen: two with extreme GC content and one with medium GC content. These families are: i. rbcL 5' UTR RNA stabilizing element (5.6% GC), ii. small nucleolar RNA SNORA67 (49.2% GC) and iii. bag-1 internal ribosome entry site - IRES (71.4% GC). The structural multiple alignments for these RNA families were downloaded from the full alignments of the Rfam database release 8.1 [11] under the Rfam accession numbers RF00197, RF00272 and RF00222, respectively. Each alignment was used to build a covariance model using the program cmbuild. These covariance models were used to score a set of random sequences, generated as described above, with length equal to the average length of the respective RNA family. This scoring was performed by the program cmsearch. Both programs, cmbuild and cmsearch, are from the package Infernal, version 0.7 [7]. Infernal embeds the null model in the covariance model. It records the emission log-odds values in the CM states instead of recording emission probabilities. Therefore, the cmsearch output score is already the log-odds score. However, this strategy can output wrong values when using a non-uniform null model. Note that the score reported by cmsearch for a sequence *x* is obtained using the best path through the Covariance Model. By definition, the best path in a CM should be the sequence of CM states that maximizes the total probability of the sequence using that path. When maximizing the log-odds score instead of the total probability, different null models may lead to a "best path" that is different from the correct one, that is, the path based only on the probability values. To circumvent this problem, we used an algebraic trick: (1) we used cmbuild to build a CM with the uniform null model *U* (that penalizes equally all nucleotides and, therefore, does not alter the best path), (2) we used this CM to score each sequence *x* (log-odds score *S_U_* and (3) we adapted the log-odds score for a given null model *N* using the equation:

*S_N_*(*x*) *= S_U_*(*x*) *+ log*(*P*(*x|U*)) *- log*(*P*(*x|N*)) (4)

Six null models were used: five fixed GC models (5%GC, 25%GC, 50%GC, 75%GC and 95%GC) and the target null model.

Default execution of cmsearch does not report hits with negative score. Since the score of most of the random sequences is negative, a small modification in cmsearch’s source code to also report negative scores was needed.

### Acceptor splice site dataset for *P. falciparum*

We have extracted 7582 acceptor splice sites from PlasmoDB release 6.4. This dataset was splitted in two parts. We have used the first part having 1000 acceptor splice sites as training set to estimate the parameters of the WAM. The second part, having 6582 acceptor splice sites, was used as positive testing set. Each acceptor splice site sequence has 70 nucleotides with the conserved AG dinucleotide at position 47.

Genomic sequences of length 70 that contains the dinucleotide AG at position 47 and were not annotated as acceptor splice sites were considered as negative samples. We have extracted a total of 939994 false acceptor splice sites as negative testing set.

### Precision Recall Graph

Since we are dealing with real data, in this experiment we used four types of null models: (i) the target null model; (ii) the genomic background null model; (iii) the uniform null model; (iv) and the training set null model.

Using these null models and the WAM estimated with the training set, we have generated the precision recall graph comparing the model in respect of different GC contents of the sequences in the testing set. We created a partition of the testing set in which each subset contains only sequences with a fixed GC content. For each subset with more than 5 positive samples, we calculated a point in the graph corresponding to the calculated precision and recall values. In this analysis we used precision  instead of specificitydue to the large number of negative samples. The recall is the same of sensitivity .

## Competing interests

The authors declare that they have no competing interests.

## Authors contributions

The data presented in the article was produced by AML and AYK. The analysis of the results and the writing of the article was performed by AML, AYK and AMD. All authors revised the final manuscript.

## Supplementary Material

Additional file 1 **ROC curve for *P. falciparum* data** This picture shows the ROC curve for the acceptor splice site prediction in *P. falciparum* comparing four different null models on respect of different GC contents of sequences. The tested null models were the target, the uniform, the training set distribution and the genomic background null models. Click here for file

## References

[B1] Henderson J Salzberg S Fasman KH Finding Genes in DNA with a Hidden Markov Model Journal of Computational Biology 1997 4 2 127 142 http://citeseer.nj.nec.com/article/henderson97finding.html 10.1089/cmb.1997.4.127 9228612

[B2] Eddy SR Multiple alignment using hidden Markov models Proceedings of the International Conference on Intelligent Systems for Molecular Biology 1995 3 114 120 7584426

[B3] Krogh A An Introduction to Hidden Markov Models for Biological Sequences 1998 In Computational Methods in Molecular Biology 45 63

[B4] Zhang MQ Marr TG A weight array method for splicing signal analysis Computer Applied in Bioscience 1993 9 499 509 10.1093/bioinformatics/9.5.499 8293321

[B5] Eddy SR Durbin R RNA sequence analysis using covariance models Nucleic Acids Research 1994 22 11 2079 2088 8029015 10.1093/nar/22.11.2079 PMC308124

[B6] Hughey R Krogh A Hidden Markov models for sequence analysis: extension and analysis of the basic method Computer Application in the Biosciences (CABIOS) 1996 12 2 95 107 10.1093/bioinformatics/12.2.95 8744772

[B7] Eddy SR A memory-efficient dynamic programming algorithm for optimal alignment of a sequence to an RNA secondary structure BMC Bioinformatics 2002 3 18 12095421 10.1186/1471-2105-3-18 PMC119854

[B8] Barrett C Hughey R Karplus K Scoring hidden Markov models Computer Applications in the Biosciences 1997 13 2 191 199 9146967 10.1093/bioinformatics/13.2.191

[B9] Deng Y Liu Q Li YX Scoring hidden Markov models Computational Biology and Chemistry 2004 28 3 189 194 10.1016/j.compbiolchem.2004.02.004 15261149

[B10] Karplus K Karchin R Shackelford G Hughey R Calibrating E-values for hidden Markov models using reverse-sequence null models Bioinformatics 2005 21 22 4107 4115 10.1093/bioinformatics/bti629 16123115

[B11] Griffiths-Jones S Moxon S Marshall M Khanna A Eddy SR Bateman A Rfam: annotating non-coding RNAs in complete genomes Nucleic Acids Research 2005 33 D121 D124 15608160 10.1093/nar/gki081 PMC540035

[B12] HMMER User Guide 2003 Ftp://selab.janelia.org/pub/software/hmmer/CURRENT/Userguide.pdf

[B13] Yu YK Wootton JC Altschul SF The compositional adjustment of amino acid substitution matrices Proceedings of the National Academy of Sciences 2003 100 26 15688 15693 10.1073/pnas.2533904100 PMC307629 14663142

[B14] Yu YK Altschul SF The construction of amino acid substitution matrices for the comparison of proteins with non-standard compositions Bioinformatics 2005 21 7 902 911 10.1093/bioinformatics/bti070 15509610

[B15] Altschul SF Madden TL Schaffer AA Zhang J Zhang Z Miller W Lipman DJ Gapped BLAST and PSI-BLAST: a new generation of protein database search programs Nucleic Acids Research 1997 25 17 3389 3402 9254694 10.1093/nar/25.17.3389 PMC146917

[B16] Schaffer AA Aravind L Madden TL Shavirin S Spouge JL Wolf YI Koonin EV Altschul SF Improving the accuracy of PSI-BLAST protein database searches with composition-based statistics and other refinements Nucleic Acids Research 2001 29 14 2994 3005 11452024 10.1093/nar/29.14.2994 PMC55814

[B17] Staden R Computer methods to locate signals in nucleic acid sequences Nucleic Acids Res 1984 12 505 519 6364039 10.1093/nar/12.1Part2.505 PMC321067

[B18] Pollastro P Rampone S HS3D, a dataset of Homo Sapiens splice regions, and its extraction procedure from a major public database International Journal of Modern Physics C 2002 13 8 1105 1117 10.1142/S0129183102003796

[B19] Kashiwabara AY MYOP: um arcabouco para predicao de genes ab initio. Master’s thesis, Universidade de Sao Paulo 2007

