# Supplementary figures and images for: Decreasing the number of false positives in sequence classification
Source: BMC Genomics. 2010 Dec 22;11(Suppl 5):S10. doi: 10.1186/1471-2164-11-S5-S10 (PMC3045793; doi:10.1186/1471-2164-11-S5-S10)

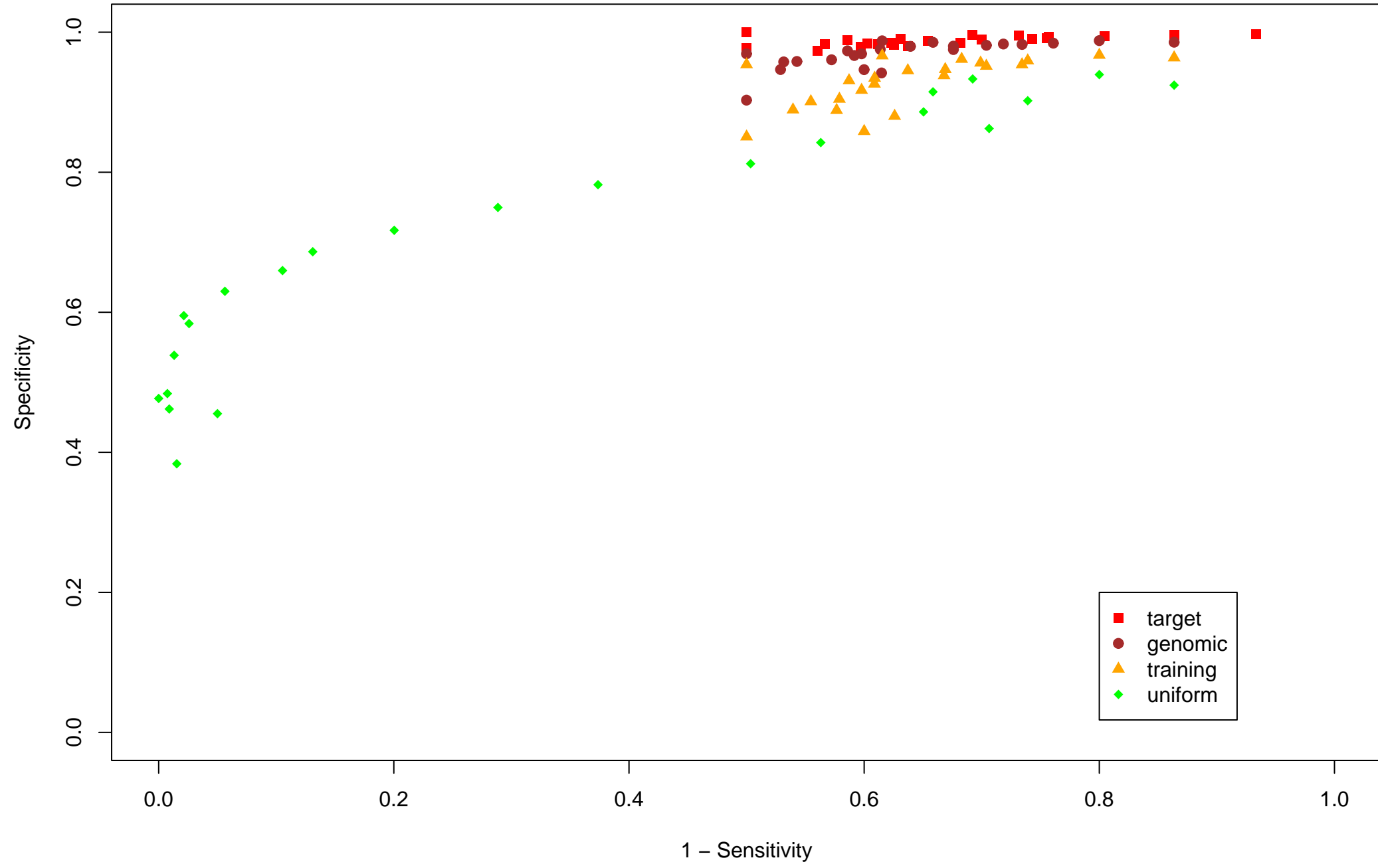

Supplement: Additional file 1 — ROC curve for P. falciparum data This picture shows the ROC curve for the acceptor splice site prediction in P. falciparum comparing four different null models on respect of different GC contents of sequences. The tested null models were the target, the uniform, the training set distribution and the genomic background null models. [file 1471-2164-11-S5-S10-S1.pdf]
